# The investigation of genetic and clinical features in patients with hereditary spastic paraplegia in central‐Southern China

**DOI:** 10.1002/mgg3.1627

**Published:** 2021-02-27

**Authors:** Chen Wang, Yun‐Jian Zhang, Ci‐Hao Xu, De Li, Zhi‐Jun Liu, Yan Wu

**Affiliations:** ^1^ Department of Neurology Wuhan Union Hospital Tongji Medical College Huazhong University of Science and Technology Wuhan China; ^2^ Department of Radiology Wuhan Union Hospital Tongji Medical College Huazhong University of Science and Technology Wuhan China; ^3^ Biobank Wuhan Union Hospital Tongji Medical College Huazhong University of Science and Technology Wuhan China

**Keywords:** *B4GALNT1*, hereditary spastic paraplegia, *SPAST*, *SPG11*

## Abstract

**Objective:**

Hereditary spastic paraplegias (HSP) is a clinically and genetically heterogeneous group of neurodegenerative disorders. We describe the genetic and clinical features of a cohort of five HSP families from central‐southern China.

**Methods:**

Using targeted exome‐sequencing technology, we investigated the genetic and clinical features in five HSP families. We reviewed the clinical histories of these patients as well as the molecular and functional characterization of the associated gene variants. We also performed functional analysis of an intron variant of *SPAST* in vitro.

**Results:**

We identified a known *SPAST* mutation (p.Pro435Leu) in a family with autosomal dominant HSP (AD‐HSP) and four novel variants in two HSP families and a sporadic case. These identified four novel variants included a variant in *SPG11* (p.Val1979Ter), two variants in *B4GALNT1* (p.Ser475Phe and c.1002 + 2 T > G), and a splicing site variant in *SPAST* (c.1245+5G>A). Minigene analysis of the splicing variant in *SPAST* (c.1245+5G>A) revealed that the mutation resulted in mRNAs with a loss of exon 9. The SPG4 family carrying c.1245+5G>A variant in *SPAST* exhibited genetic anticipation, with a decreased age at onset and increased severity in successive generations. The proband with p.Val1979Ter variant in *SPG11* showed characteristic clinical features of early‐onset, severe spasticity, and corpus callosum atrophy which were highly suggestive of the diagnosis of SPG11‐associated HSP.

**Conclusions:**

Our findings strongly support variable phenotype of *B4GALNT1*‐related SPG26 and also expand the clinical and mutation spectrum of HSP caused by mutations in *SPAST*, *SPG11*, and *B4GALNT1*. These results will help to improve the efficiency of early diagnosis in patients clinically suspected of HSP.

## Abstract 2

We investigated the genetic and clinical features in five families with HSP from central‐southern China using targeted exome‐sequencing technology. We identified a known mutation (p.Pro435Leu) in *SPAST* in a family with autosomal dominant HSP (AD‐HSP) and four novel variants in three independent HSP families and a sporadic case. These identified four novel variants include a nonsense variant (p.Val1979Ter) in *SPG11*, two variants (p.Ser475Phe and c.1002 + 2 T > G) in *B4GALNT1*, and a splicing site variants in *SPAST* (c.1245 + 5G>A). Minigene analysis of the splicing variant (c.1245 + 5G>A) in *SPAST* revealed that the mutation resulted in mRNAs with a loss of exon 9

## 1 INTRODUCTION

Hereditary spastic paraplegias (HSP) is a heterogeneous group of genetically determined neurodegenerative disorders characterized by progressive weakness and spasticity in the lower limbs. Clinically, HSP has been divided into pure and complicated forms, based on the manifestation of additional features, such as ataxia, intellectual disability, dysarthria, extrapyramidal disturbance, and peripheral neuropathy. The disease can occur at any age, but it most commonly affects children between 3 and 15 years. It affects individuals of diverse ethnic groups with significantly variable prevalence estimates ranging from 2 to 10 per 100,000 (Ruano et al., 2014).

HSP can be inherited in an autosomal dominant (AD‐HSP), autosomal recessive (AR‐HSP), X‐linked manner (XL‐HSP), as well as mitochondrial maternal transmission. To date, more than 80 genes or sites have been identified, of which *SPAST* (previously known as *SPG4*; OMIM:604277) and *SPG11* (OMIM:610844) appear to be the most common genetic cause of the AD‐HSP and AR‐HSP, respectively (Boutry et al., 2019; Klebe et al., 2015).

We have recently performed comprehensive genetic investigations in Chinese HSP patients and also have found that SPG4 and SPG11 were the most frequent forms of AD‐HSP and AR‐HSP in southeast China, respectively (Lu et al. 2018; Wei et al., 2019). However, there are still very few reports on genetic analysis of HSP patients in central‐southern China. In this study, we conducted a targeted exome‐sequencing in five independent families with HSP from central‐southern region of China, to enrich the profile of genetic and clinical features of Chinese HSP patients. To the best of our knowledge, this is the first report of mutation in *B4GALN*T1 (OMIM:601873), also known as *SPG26*, in Chinese patient with HSP. Being able to define the genetic and clinical profile of SPG26‐associated HSP may improve understanding in disease heterogeneity and severity.

## 2 MATERIALS AND METHODS

### 2.1 Subjects

Thirteen patients from five unrelated Chinese HSP pedigrees, including five probands and eight affected family members, were recruited for this study. All the patients were evaluated by at least two senior neurologists and diagnosed according to Harding's criteria. They are mainly from central‐southern region of China (Han Chinese) and collected from the Neurology Department of Wuhan Union hospital, between September 2017 and October 2019. DNA samples were obtained from all the subjects, including the probands, as well as affected and unaffected family members. Blood biochemical analysis, electromyogram (EMG) and Nerve conduction tests (NCT), and magnetic resonance imaging (MRI) of brain and spinal cord were conducted. An additional 200 normal individuals were included as controls after exclusion of the occurrence of common neurological disorders. Written informed consents were obtained from all participants. This study was approved by the ethics committee of Wuhan Union Hospital (Reference number: 2019‐S1136).

### 2.2 Genetic analysis

Genomic DNA was extracted from peripheral blood samples using Blood Genomic Extraction Kit (Qiagen). A panel was designed to cover 261 genes, including 67 known genes responsible for dominant and recessive HSP forms and other 194 genes associated with spastic paraplegia (Table S1). Deep sequencing was performed using Illumina Hiseq2000 system (GrandOmics Biosciences Co). The annotation and analysis of sequenced reads were performed as described previously (Liu et al., 2019). Sanger sequencing was used to validate the candidate variants after data analysis. Co‐segregation analysis was conducted through screening for the confirmed variants in the family members. To further screen for large deletions or duplications of culprit genes (*SPAST*, *ATL1*, *REEP1*, *PGN*, and *SPG11*), the Multiplex ligation‐dependent probe amplification assay (MLPA) analysis was performed as we described previously (Lu et al., 2018). Moreover, analysis of trinucleotide repeats in SCA 1, 2, 3, 6, 7, 12, and 17, as well as Dentatorubral–pallidoluysian atrophy (DRPLA), which could not be detected by targeted sequencing analysis, was conducted via repeat‐primed PCR combined with fragment length analysis.

### 2.3 Minigene analysis

Construction of minigene was based on the pSPL3 exon trapping vector (Invitrogen). The *SPAST* exon relevant to the splicing variant (c.1245+5G>A) along with flanking intronic sequences was amplified by PCR of genomic DNA from the patient and control, with primers containing restriction sites for XhoI and EcoRI (5′‐GGTACGGGATCACCAGAATTCCACCTGGCCTCATAGCTTAC‐3′ (forward) and 5′‐ATCCTGCAGCGGCCGCTCGAGCTGATGTTTAAGCCAGCCAG‐3′ (reverse)). The PCR products were then cloned into the splicing reporter pSPL3 vector and transfected into HEK293 T cells. The RNA extraction and RT‐PCR were performed using the RNAiso Plus and PrimeScript RT reagent kit (Takara), respectively. Electrophoresis and sanger sequencing were used for splicing pattern analysis.

## 3 RESULTS

### 3.1 Clinical manifestations of probands

**FIGURE 1:**
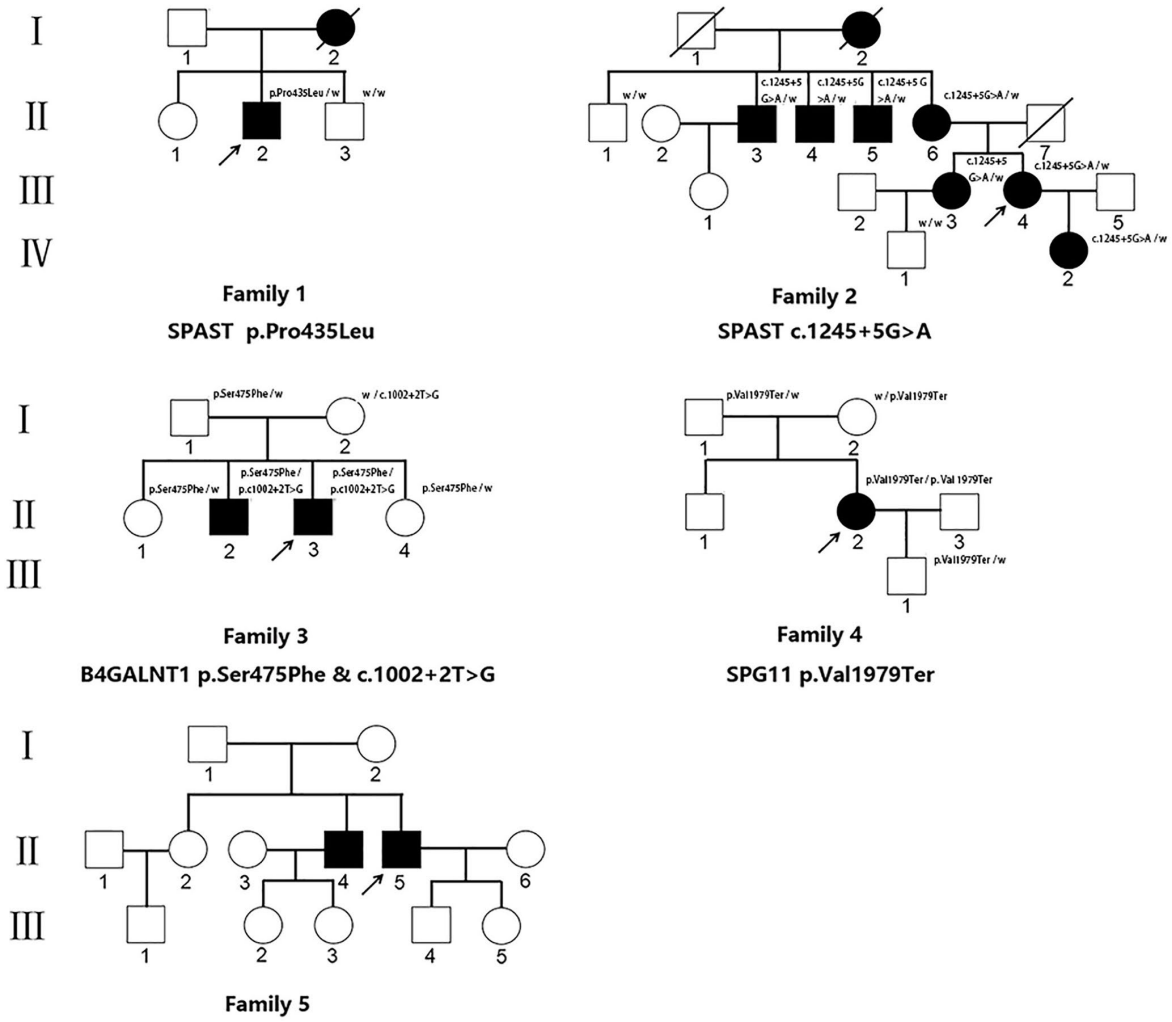
Pedigrees of five HSP patients in present study. Squares indicate males; circles indicate females; filled symbols indicate affected individuals; diagonal lines across symbols indicate deceased individuals; arrows indicate the probands; “w” indicate the wild type allele.

The clinical manifestations of all probands were summarized in Table 1, and pedigrees of the families were displayed in Figure 1. Sampled subjects were marked with genotype in Figure 1 as well. In total, we recruited 13 affected individuals from 5 independent families, including eight males and five females. The mean age at onset was 30.23 years, ranging from 7 to 48 years. Among the five probands, two showed AD inheritance pattern, three with AR inheritance pattern. Apart from the most common symptoms of progressive lower limbs weakness and spasticity, additional presentations including dysarthria, dysphagia, urinary incontinence, constipation, and intelligence impairment were also observed in present study.

**TABLE 1:** Clinical presentations and genetic analysis results of 5 HSP probands

Proband no	Sex	AAO (y)	Inheritance	LL weakness	LL plasticity	Reflexia	Intellectual disability	EMG/NCT	Brain MRI	Additional features	Gene	Pathogenic variants	ACMG criteria
1	F	48	AD	+	+	++++	–	normal	normal	dysarthria	*SPAST*	c.1304C>T (p. Pro435Leu)	Likely pathogenic
2	M	26	AD	+	+	++++	–	normal	normal	constipation	*SPAST*	c.1245+5G>A	Likely pathogenic
3	M	7	AR	+	+	++++	+	normal	normal	poor social communication skills	*B4GALNT1*	c.1424C>T (p. Ser475Phe)	Likely pathogenic
												c.1002 + 2 T > G	Likely pathogenic
4	M	12	AR	+	+	+++	+	normal	thin corpus callosum	urinary dysfunction, dysarthria, dysphagia	*SPG11*	c.5934_5935insTAACCTGGAA (p. Val1979Ter)	Likely pathogenic
5	M	41	AR	+	+	++++	–	normal	normal	—	—	—	—

—, absent; AAO, age at onset; AD, autosomal dominant; AR, autosomal recessive; EMG, electroneuromyography; F, female; LL, lower limb; M, male; MRI, Magnetic Resonance Imaging; NCT, nerve conduction test.

### 3.2 Genetic findings

**FIGURE 2:**
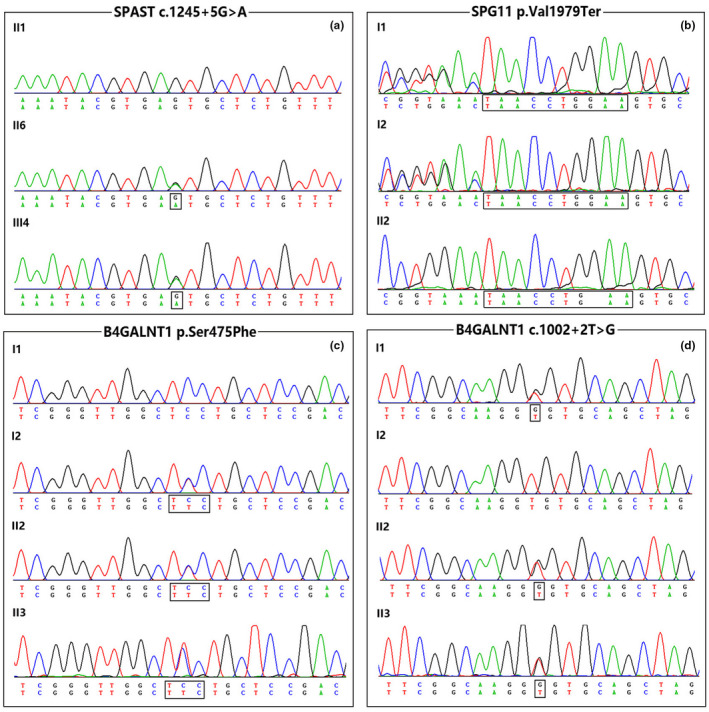
Chromatograms of four novel variants identified in *SPAST*, *SPG11*, and *B4GALNT1* gene, respectively. (a) The c.1245+5G>A variant in *SPAST* from family 2. (b) The p. Val1979Ter variant in *SPG11* from family 4. (c) The p. Ser475Phe variant in *B4GALNT1* from family 3. (d) The c.1002 + 2 T > G variant in *B4GALNT1* from family 3.

In general, about 97.5% of the target bases were covered with at least 50X per individual, and the mean depth of coverage for all target regions was 185. After filtering and validation by Sanger sequencing, 5 probable pathogenic variants were identified in 4 probands, involving one variant previously reported as pathogenic variant and four novel variants. The known pathogenic variant c.1304C>T (p. Pro435Leu) was identified in *SPAST* (NM_014946; Magariello et al., 2006). The identified four novel variants include two variants (c.1424C>T[p. Ser475Phe] and c.1002 + 2 T > G) in *B4GALNT1* (NM_001478), a frameshift variant (c.5934_5935 ins TAACCTGGAA[p. Val1979Ter]) in *SPG11* (NM_025137), and a splicing site variants (c.1245+5G>A) in *SPAST* (Figure 2). All of these variants, being predicted as harmful effects by the SIFT, PolyPhen‐2, dbscSNV and Human Splicing Finder (HSF) software, were absent in publicly available database as well as 200 normal controls. According to the American College of Medical Genetics and Genomics (ACMG) standards and guidelines (Richards et al., 2015), all four variants were classified as likely pathogenic variants. The novel splicing mutation (c.1245+5G>A) in *SPAST* was co‐segregated with the phenotypes observed in family 2. Moreover, pathogenic repeat expansions which are implicated in all SCA types (SCA types 1–3, 6, 7, 12, and 17) and DRPLA, as well as large deletions or duplications of HSP culprit genes (*SPAST*, *ATL1*, *REEP1*, *PGN*, and *SPG11*), was excluded in this family. The two novel variants in *B4GALNT1* identified in compound heterozygous state, including a splicing mutation site (c.1002 + 2 T > G) and a missense mutation (p. Ser475Phe), were found to segregate with the disease in family 3. In family 4, co‐segregation analysis showed that the father and mother are both heterozygous for the novel stop‐gained *SPG11* variant (p. Val1979Ter). In family 5, no mutation was found in the DNA sample of proband who was the only sequenced family member.

### 3.3 Splicing pattern analysis

**FIGURE 3:**
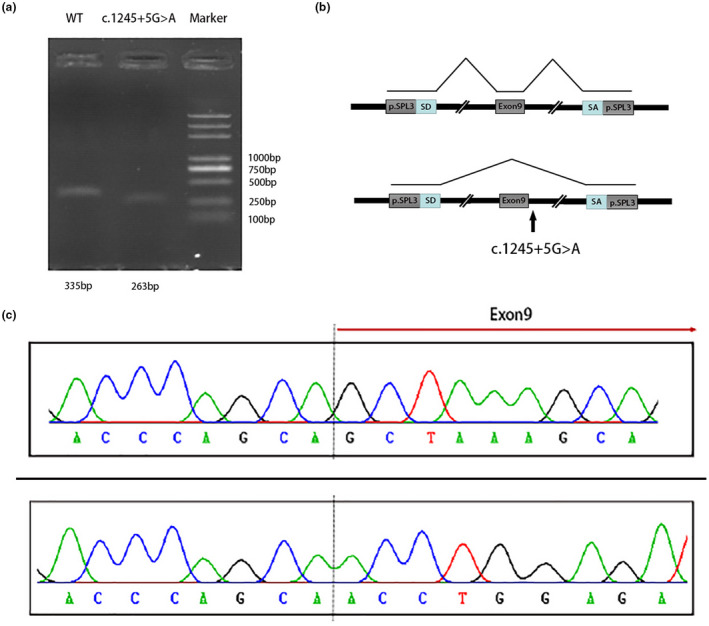
Results of minigene splicing assay for the *SPAST* c.1245+5G>A variant. (a) cDNA products were separated by agarose gel electrophoresis. Lane1: WT [263 bp +72 bp (exon 9)]; Lane2: c.1245+5G>A variant [263 bp]; Lane3: DNA Marker. (b) Schematic diagram of splicing reporter minigene construction. Splice donor (SD) and splice acceptor (SA) are two exons of the pSPL3 vector. (c) The sequencing results for the two bands after electrophoresis. The upper chromatogram: 335 bp; The bottom chromatogram: 263 bp.

A splicing reporter minigene with *SPAST* exon 9 and mutant intron was constructed. Agarose gel electrophoresis of RT‐PCR amplified products showed that the fragment obtained in mutant group (263 bp) was smaller than that obtained in control group (335 bp) (Figure 3a). The upper chromatogram in Figure 3b illustrated the normal splicing pattern of wild type while the bottom panel showed the abnormal splicing pattern of mutant sequence. Sanger sequencing showed that the c.1245+5G>A mutation in *SPAST* resulted in aberrant mRNAs with a loss of exon 9 (Figure 3c).

### 3.4 Characteristics of HSP patients with *SPAST* mutations

The proband (**II:2**) in family 1, carrying p. Pro435Leu variant in *SPAST*, was a 56‐year‐old male and presented with lower limbs weakness and gait disturbance at the age of 48. As the disease progresses, he began to show dysarthria and severe spasticity in the lower limbs. He currently needs to move around with the aid of crutch. No signs of intellectual disability were observed in the proband. Neurological examination showed hyperactive deep tendon reflexes in the lower limbs, gait ataxia, pes cavus, and positive Babinski signs. The EMG results and Brain MRI were unremarkable. His mother (I:2) developed the illness at the age of 50 with a disease duration of 9 years.

The proband (III:4) in family 2, a 36‐year‐old female, was detected as the carrier of c.1245+5G>A variant in *SPAST*. She presented with lower limbs weakness and an unsteady gait at the age of 26. As the disease progressed, her gait disturbance became more severe with frequent falling. She had constipation problems since age 31. Severe spasticity was marked in the lower limbs although the early‐stage of baclofen treatment which was clinically used to relieve limb spasticity was taken to the proband. Her examination was further marked by hyperactive deep tendon reflexes and positive Babinski signs, with mild rigidity in all extremities especially in the lower limbs. She manifested bilateral pes cavus and ankle clonus. Her older sister (III:3) experienced the similar symptoms at the age of 26. The proband's mother (II:6) and other three affected uncles (II:3, III:4, III:5) presented with gait disturbance or walking difficulty at the age of 42, 44, 45 and 44, respectively. The proband's grandmother (I:2) was diagnosed with HSP when she was 50 years old and died at the age of 70 years. The proband's daughter (**IIII:2**) had initial symptom of gait disturbance at age 7 years, and experienced mild cognitive impairment, with a Mini‐Mental State Exam (MMSE) score of 22/30, eight years after symptom onset. She had poor concentration and social communication skills. No muscular atrophy or fasciculation was observed in all affected individuals within this family. Upon neurological examination, these patients revealed moderate or severe spasticity in the lower limbs, pyramidal‐tract signs, and gait ataxia. The Brain and spinal cord MRI were normal. The EMG and nerve conduction test were also unremarkable. In this family, the mean age of onset of HSP was 50, 44, 26, and 7 years in members of the first, second, third, and fourth generations of this family, respectively. It is noteworthy that the age at onset was decreased with each subsequent generation (*p* < 0.05). Remarkably, the only patient (IIII:2) in the fourth generation was younger at onset and had a more severe gait disturbance accompanied by mental impairment than the previous generations.

### 3.5 Presentations of HSP patients with *B4GALNT1* mutations

The proband (**II:3**) from family 3 carrying c.1002 + 2 T > G and c.1424C>T (p. Ser475Phe) variants in *B4GALNT1* presented with subtle gait abnormalities at the age of 7 years. His gait disturbance was gradually observed as disease progresses, when he began to experience severe spasticity in the lower limbs. He had learning disability with poor academic performance and social communication skills. Muscle weakness and atrophy were not obviously observed in proximal limbs, but with a slightly reduced power of 4 to 5/5 and atrophy in the distal areas. His sensory, cerebellar, and sphincter function were not affected. Neurological examination at the age of 15 years showed severe spasticity in the lower limbs, exaggerated reflexes, pes cavus and pyramidal tract signs. Cerebral and spinal MRI showed no significant changes in the brain and spinal cord. Nerve conduction tests and EMG were unremarkable. His older brother (II:2), aged 28 years old, experienced the similar symptoms at the age of 8 years. He developed gait abnormality and severe lower limb spasticity. He is currently ambulatory with wheelchair assistance. In addition, he was unable to interact normally with others, and his cognitive function declined significantly, with a MMSE score of 5/30. Examination showed severe spasticity and weakness in the lower limbs, severe muscle atrophy involving the whole body, exaggerated reflexes, pes cavus and severe pyramidal tract signs. Consanguinity was not reported in the family.

### 3.6 Characteristics of HSP patients with *SPG11* mutation

The proband (II:2) from family 4 carrying the homozygous mutation (p. Val1979Ter) in *SPG11* initially experienced gait disturbance at the age of 12 years. She showed an ataxic gait and frequently fell down. Three years after these symptoms, she complained progressive lower limb weakness, dysarthria, dysphagia, intellectual disability and urinary dysfunction occasionally. She was wheelchair‐bound by April 2018. Neurological examination showed spasticity in the lower limbs, atxia gait, increased muscle tone, hyperactive deep tendon reflexes, and positive Babinski signs. No muscular atrophy or fasciculation was observed. Brain MRI showed an abnormally thin corpus callosum. Nerve conduction tests and EMG revealed no evidence of neurological disturbance. No history of neurologic disease was found in this family. It is noteworthy that her parents were non‐consanguineous.

## 4 DISCUSSION

Using targeted exome‐sequencing technology, we investigated the profile of genes mutated and clinical features in five unrelated HSP families in central‐southern China. We detected a known *SPAST* p. Pro435Leu mutation and four novel likely pathogenic variants including two variants in *B4GALNT1* (p. Ser475Phe and c.1002 + 2 T > G), a p. Val1979Ter variant in *SPG11*, and an intronic variant in *SPAST* (c.1245+5G>A). All of these novel variants were described for the first time. No causative variants were found in family 5, which suggests that whole‐exome or whole‐genome sequencing should be further performed to explore the new potential genes associated with the disease. We also performed in vitro splicing pattern analysis of intronic variant c.1245+5G>A in *SPAST*. There is a known pathogenic variant c.1245+4A>G reported with only one nucleotide difference, which could produce both normal‐length and truncated transcripts in patient‐derived RNA and in vitro splicing analysis (Svenson, Ashley‐Koch, Gaskell, et al., 2001). This variant associated with low penetrance was considered to be “leaky splicing” involve splicing mutations in the *SPAST* gene (Svenson et al., 2001). In present study, minigene analysis of the splicing variant (c.1245+5G>A) in *SPAST* revealed that the variant resulted in only truncated mRNA transcripts with a loss of exon 9. Due to the importance of genomic‐sequence context in the determination of splice‐site selection demonstrated in further study and limitation of our minigene structure design, in vitro splicing analysis of minigene constructs including expanded flanking sequence need to be further studied to verify the presence of leaky splicing.

SPG4‐associated HSP may exhibit high interfamilial and intrafamilial phenotypic variability including age at onset and disease severity (Tesson et al., 2015). Genetic anticipation has been reported in many SPG4‐associated HSP families (Kawarai et al., 2017; Lan et al., 2012; Reddy et al., 2007; Rodrigues et al., 2020). None of pathogenic trinucleotide‐repeats expansion, often responsible for genetic anticipation of repeat expansion diseases, was described in SPG4 or other types HSPs. In line with previous study, the SPG4 family in present study showed genetic anticipation, with a decreased age at onset and increased severity in successive generations. In addition to SPG4, genetic anticipation has also been reported in other types of HPS, such as SPG3 and SPG31 (Kamada et al., 2018; Ming, 2007; Rodrigues et al., 2020). There may be other environmental and genetic modifiers influencing phenotype variability. An epistatic effect between *DPY30* and *SPAST* was previously reported, which affects age at onset in a cohort of SPG4‐related HSP patients (Newton et al., 2018). Although none of known pathogenic repeat expansions was found in Family 2, the possibility of repeat expansion in novel loci needs to be explored by new technology (Dolzhenko et al., 2020; Trost et al., 2020) which was developed to detect genome‐wide repeat expansions.

SPG26‐related HSP cases have been reported worldwide but mainly distribute in Europe, South America, and North America. In total, 13 pathogenic variants of *B4GALNT1*, including a splicing mutation, 7 missense mutations and 5 inserts/deletions mutations, have already been reported in 14 SPG26‐related HSP families with variable complicated phenotypes (Boukhris et al., 2013; Dad et al., 2017; Harlalka et al., 2013; Rose et al., 2020; Wakil et al., 2014). To the best of our knowledge, this is the first description of *B4GALNT1* mutations in Chinese patient with HSP. Mutations in *B4GALNT1* were also reported to in relation to autism spectrum disorder as well as cerebellar ataxia (Fogel et al., 2014; Lossifov et al., 2014). The clinical features of all published SPG26 cases with *B4GALNT1* mutation are summarized in Table S1. Altogether, patients carrying *B4GALNT1* mutation has a mean age at onset of 9.15 years, ranging from 1.3 to 39 years. In line with previous studies, our findings strongly support complicated phenotypic features of SPG26‐related HSP characterized by slowly progressive lower limbs spasticity, early‐onset, mental retardation, cognitive impairment, and extrapyramidal features. None of evidence of peripheral neuropathy was observed in present case. Therefore, *B4GALNT1* mutation should be explored in AR‐HSP patients with early age at onset and intellectual deficit. B4GALNT1 is a Golgi‐residing enzyme with type II membrane topology and its mutation may result in a deficiency of GM2/GD2 synthase. The specific role of GM2/GD2 synthase deficiency in complicated phenotype of HSP remains unclear and further studies are needed to elucidate the mechanism.

*SPG11* is the most common cause of AR‐HSP and more than 36 mutations in *SPG11* have already been reported in Chinese HSP patients. In this study we identified a novel homozygous insertion mutation (p. Val1979Ter) in *SPG11* gene which resulted in premature protein termination. This variant was considered as pathogenic variant based on its predicted impact on SPG11 protein. This mutation segregated with the disease in this pedigree as the proband carried homozygous mutation and his unaffected parents carried heterozygous mutation. SPG11‐associated HSP are often characterized by early‐onset, lower limbs spasticity, dysarthria, cognitive decline, peripheral neuropathy, and thin corpus callosum (Pensato et al., 2014; Stevanin et al., 2008). In the present case, although no sign of cognitive impairment and peripheral neuropathy was observed, the characteristic clinical feature of early‐onset, severe spasticity, and corpus callosum atrophy are highly suggestive of the diagnosis of SPG11‐associated HSP.

## 5 CONCLUSION

We detected a known disease‐causing variant in *SPAST* gene and four novel likely pathogenic variants in *SPAST*, *SPG11*, and *B4GALNT*, respectively. Our findings expand the clinical and mutation spectrum of HSP caused by mutations in these genes. These results will help improve the efficiency of early diagnosis in patients clinically suspected of HSP.

## CONFLICTS OF INTEREST

The authors declare no conflict of interest.

## AUTHORS’ CONTRIBUTIONS

WC, ZYJ, and LZJ conceived and designed the content of the paper. WC and ZYJ were involved in data collection, data interpretation, and contributed equally to the first draft. XCH and LD contributed to literature search. WC, ZYJ, XCH, and LD wrote the manuscript. LZJ and WY revised the paper and all authors read and approved the manuscript.

## Supporting information

Table S1

Table S2

## Data Availability

All data used for analysis are shown in the figures and tables in this article. Data sharing is applicable to this article if requested by other investigations for purposes of replicating the results.
