# Supplementary material for: The investigation of genetic and clinical features in patients with hereditary spastic paraplegia in central‐Southern China
Source: Mol Genet Genomic Med. 2021 Feb 27;9(5):e1627. doi: 10.1002/mgg3.1627 (PMC8172193; doi:10.1002/mgg3.1627)
Supplement: Supplementary file 2 — Table S2 [file MGG3-9-e1627-s001.doc]

**Supplementary table 2. Summary of clinical manifestations of all published cases with *B4GALNT1* mutations**

| Patient  no | Sex | AAO  (y) | Geographical  Origin | Intellectual disability | LL plasticity | LL reflexes | LL weakness | Muscle  wasting | Urinary  symptom | Pes cavus | Additional features | EMG/NCT | Brain MRI | Mutation |
| --- | --- | --- | --- | --- | --- | --- | --- | --- | --- | --- | --- | --- | --- | --- |
| 1 | M | 2 | Portugal | mild | moderate | - | severe | severe | yes | severe | dyskinesia | - | - | c.358C>T (p.Q120*) |
| 2 | M | 2 | Portugal | mild | moderate | + | severe | severe | yes | severe | dyskinesia | - | - | c.358C>T (p.Q120*) |
| 3 | M | 3 | Portugal | mild | moderate | + | moderate | moderate | no | severe | no | normal | cerebral atrophy | c.358C>T (p.Q120*) |
| 4 | F | 2 | Portugal | mild | mild | - | moderate | moderate | yes | severe | dyskinesia | - | normal | c.358C>T (p.Q120*) |
| 5 | M | 8 | Spain | mild | yes | +++ | - | yes | no | yes | cataract, diplopia | ASN | temporal atrophy | c.395delC (p.P132Qfs*7) |
| 6 | F | 8 | Spain | mild | yes | +++ | - | yes | yes | yes | cataract | ASN | frontal and temporal atrophy | c.395delC (p.P132Qfs*7) |
| 7 | M | 7 | Spain | mild | yes | - | - | yes | yes | - | cataract, diplopia | - | - | c.395delC (p.P132Qfs*7) |
| 8 | M | 9 | Brazil | mild | moderate | ++++ | mild | no | no | no | strabismus, dysmorphic, glaucoma | normal | normal | c.682C>T (p.R228*) |
| 9 | F | 14 | Brazil | mild | severe | ++++ | moderate | no | no | yes | strabismus, dysmorphic | normal | normal | c.682C>T (p.R228*) |
| 10 | M | 19 | Tunisia | mild | severe | ++++ | moderate | no | no | no | no | normal | normal | c.898C>T (p.R300C) |
| 11 | M | 3 | Tunisia | mild | severe | ++++ | severe | severe | yes | yes | dyskinesia | - | - | c.898C>T (p.R300C) |
| 12 | F | 10 | Tunisia | mild | moderate | +++ | no | no | no | no | no | - | - | c.898C>T (p.R300C) |
| 13 | F | 15 | Tunisia | mild | moderate | +++ | mild | no | no | no | no | - | - | c.898C>T (p.R300C) |
| 14 | M | 4 | France | moderate | severe | - | moderate | severe | no | yes | dystonia, tremor, dysphagia | ASN | cerebral atrophy | c.918_923dupTACCGT (p.T307_V308dup) &  c.1315_1317dupTTC(p.F439del) |
| 15 | M | EC | German | moderate | severe | - | moderate | yes | no | no | depressive episode, dystonia | AMN | - | c.1298A>C (p.D433A) |
| 16 | M | EC | German | moderate | moderate | - | severe | no | no | no | depressive episode, dystonia | AMN | - | c.1298A>C (p.D433A) |
| 17 | M | EC | German | moderate | no(initially severe) | - | severe | yes | no | no | organic psychosis, dystonia, strabismus | AMN | cerebral atrophy | c.1298A>C (p.D433A) |
| 18 | M | 11 | Algeria | moderate | moderate | +++ | moderate | no | no | no | strabismus, ocular telangiectasia | ASN | cerebral atrophy | c.263dupG (p.L89Pfs*13) |
| 19 | F | 6 | Kuwaiti | mild | moderate | +++ | - | severe | - | - | dysarthria, tongue tremors, emotional lability | normal | normal | c.1458dupA(p.L487Tfs*77) |
| 20 | F | 6 | Kuwaiti | no | moderate | +++ | - | mild | - | - | dysarthria, tongue tremors, emotional | normal | normal | c.1458dupA(p.L487Tfs*77) |
| 21 | M | 6 | Kuwaiti | mild | moderate | +++ | - | severe | - | - | dysarthria, tongue tremors, emotional | normal | normal | c.1458dupA(p.L487Tfs*77) |
| 22 | F | 6 | Kuwaiti | mild | moderate | +++ | - | severe | - | - | dysarthria, tongue tremors, emotional | normal | normal | c.1458dupA(p.L487Tfs*77) |
| 23 | F | 11 | Kuwaiti | no | moderate | - | - | severe | - | - | dysarthria, tongue tremors, emotional | normal | normal | c.1458dupA(p.L487Tfs*77) |
| 24 | M | 37 | Italian-Canadian | no | moderate | ++ | yes | no | - | no | vestibular hypofunction | ASN | normal | c.852G>C (p.K284N) |
| 25 | F | 39 | Italian-Canadian | no | moderate | ++ | yes | no | - | no | psychiatric illness | - | normal | c.852G>C (p.K284N) |
| 26 | F | 1.6 | Amish | severe | moderate | ++ | - | yes | - | yes | depression and psychotic features | - | - | c.1514G>A (p.R505H) |
| 27 | M | EC | Amish | mild | moderate | ++ | yes | mild | no | yes | seizure, autistic, cataracts | - | - | c.1514G>A (p.R505H) |
| 28 | F | EC | Amish | mild | moderate | ++ | yes | mild | - | yes | seizure, dysmetria | - | - | c.1514G>A (p.R505H) |
| 29 | M | 1.3 | Amish | moderate | moderate | +++ | no | - | - | no | poor concentration and social communication skills | - | - | c.1514G>A (p.R505H) |
| 30 | - | EC | Bedouin | moderate | severe | +++ | - | - | - | - | autistic, paranoia, social phobia | - | - | c.1003-2A > G |
| 31 | - | 6-7 | Bedouin | mild | moderate | ++ | - | yes | - | - | tongue tremors, dysarthria | - | normal | c.1458dupA(p.L487fs) |
| 32 | F | 8 | Saudi | normal | mild | ++ | - | no | - | - | no | normal | - | c.C1358G>A (p.P453R) |
| 33 | M | 5 | Saudi | normal | moderate | ++ | - | no | - | - | dysarthria | AMN | - | c.C1358G>A (p.P453R) |
| 34 | F | EC | Mexico | severe | moderate | ++ | yes | yes | - | yes | epilepsy, autism, psychiatric  illness | - | - | c.263dupG(p.L89Pfs*13) |
| 35 | M | 7 | China | no | severe | ++++ | yes | no | no | yes | no | normal | normal | c.1002+2T>G & c.1424C>T(p.S475F) |

F, female. M, male. AAO, age at onset. y, year. EC, early childhood. LL, lower limb. AMN, axonal motor neuropathy. ASN, axonal sensory neuropathy. EMG, electroneuromyography. NCS, nerve conduction test. -, absent.
